# Crosstalk of cell death pathways unveils an autophagy-related gene AOC3 as a critical prognostic marker in colorectal cancer

**DOI:** 10.1038/s42003-024-05980-6

**Published:** 2024-03-09

**Authors:** Hui Xu, Haiyang Cui, Siyuan Weng, Yuyuan Zhang, Libo Wang, Zhe Xing, Xinwei Han, Zaoqu Liu

**Affiliations:** 1 Department of Interventional Radiology, The First Affiliated Hospital of Zhengzhou University, Zhengzhou, Henan 450052 China; 2 Interventional Institute of Zhengzhou University, Zhengzhou, Henan 450052 China; 3 Interventional Treatment and Clinical Research Center of Henan Province, Zhengzhou, Henan 450052 China; 4 Department of Radiology, The First Affiliated Hospital of Zhengzhou University, Zhengzhou, Henan 450052 China; 5 Department of Hepatobiliary and Pancreatic Surgery, The First Affiliated Hospital of Zhengzhou University, Zhengzhou, Henan China; 6 Department of Neurosurgery, The Fifth Affiliated Hospital of Zhengzhou University, Zhengzhou, China; 7 Institute of Basic Medical Sciences, Chinese Academy of Medical Sciences and Peking Union Medical College, Beijing, 100730 China

**Keywords:** Classification and taxonomy, Tumour heterogeneity, Colorectal cancer

## Abstract

The intricate crosstalk of various cell death forms was recently implicated in cancers, laying a foundation for exploring the association between cell death and cancers. Recent evidence has demonstrated that biological networks outperform snapshot gene expression profiles at discovering promising biomarkers or heterogenous molecular subtypes across different cancer types. In order to investigate the behavioral patterns of cell death-related interaction perturbation in colorectal cancer (CRC), this study constructed the interaction-perturbation network with 11 cell death pathways and delineated four cell death network (CDN) derived heterogeneous subtypes (CDN1-4) with distinct molecular characteristics and clinical outcomes. Specifically, we identified a subtype (CDN4) endowed with high autophagy activity and the worst prognosis. Furthermore, *AOC3* was identified as a potential autophagy-related biomarker, which demonstrated exceptional predictive performance for CDN4 and significant prognostic value. Overall, this study sheds light on the complex interplay of various cell death forms and reveals an autophagy-related gene *AOC3* as a critical prognostic marker in CRC.

## Abstract (web summary)

In colorectal cancer, four cell death subtypes (CDN1-4) are identified, each with distinct traits and prognoses.

## Introduction

As the second most deadly worldwide, colorectal cancer (CRC) is featured by dramatical heterogeneity and invasiveness^1^. By 2030, the global burden of CRC is expected to increase by 60%, with more than 1.1 million deaths and 2.2 million new cases^2^, demonstrating the imperative to improve the diagnosis and treatment of CRC patients. With advances in high-throughput sequencing and experimental techniques, numerous molecular biomarkers and taxonomies have been developed to facilitate the clinical management of CRC. A multicenter study of 13 countries internationally validated an Immunoscore measured by the abundance of CD3+ and CD8 + T cells in the center and invasive margin of tumors, which could accurately estimate the recurrence risk of CRC patients^3^. Our study previously integrated ten machine-learning algorithms to construct a multi-gene panel for improving outcomes in CRC^4^. Moreover, molecular subtypes based on snapshot gene expression or multi-omics data have represented a tremendous stride forward in deciphering intertumoral heterogeneity and optimizing individual treatments of CRC^5–9^, such as Consensus Molecular Subtypes (CMS)^5^ and CRC Intrinsic Subtypes (CRIS)^6^. Nonetheless, existing tools were commonly developed based on the expression profiles of multiple genes, obviously ignoring the biological interactions across various genes within a defined pathway^10–12^.

Recently, various patterns of cell death have been investigated with each profoundly impacting the initiation and development of cancers^13^. In CRC, unbalanced or defective cell death signaling is involved in the pathogenesis of colorectal diseases ranging from chronic bowel disease to colorectal cancer^14^. Additionally, cell death pathways are essential targets for current therapies against CRC. For example, inhibition of the autophagy pathway has been proven to reduce the proliferative ability of *RAS*-driven CRC^15^. Chaudhary et al. suggested that *LCN2* overexpression could lead to 5-fluorouracil resistance via suppressing ferroptosis in CRC^16^. More importantly, emerging evidence casts an increasingly clear point that these pathways tend to operate together. For example, ferroptosis and necroptosis can be triggered by shared stimuli and are both involved in ischemia-reperfusion driven pathologies^13^. Autophagy has been implicated as a backup mechanism for apoptosis to play a common role in reducing malformations to maintain normal growth and development^17^. Nonetheless, little is known about the impact of the crosstalk of different cell death forms on molecular heterogeneity in CRC.

Broad high-throughput data bring opportunities to integratively explore various patterns of cell death. Here, we retrieved 11 cell death pathways to constitute the interplay network as previously reported^12^. As is well-known, biological networks tend to maintain stable in normal tissues, but are significantly perturbated in cancer tissues^12,18^. Hence, this study initially constructed the perturbation matrix of 11 cell death pathways based on the expression profiles of CRC tissues and normal colon tissues. Subsequently, four cell death-related network (CDN) subtypes were deciphered from the perturbation matrix, which displayed considerable heterogeneity in clinical outcomes, molecular characteristics, and biological processes. Notably, *AOC3* was identified as an autophagy-related biomarker predictive of CDN4 subtype, which could be an important prognostic marker in colorectal cancer.

## Results

### Calculation of interaction-perturbation in the cell death interplay network

**Fig. 1:**
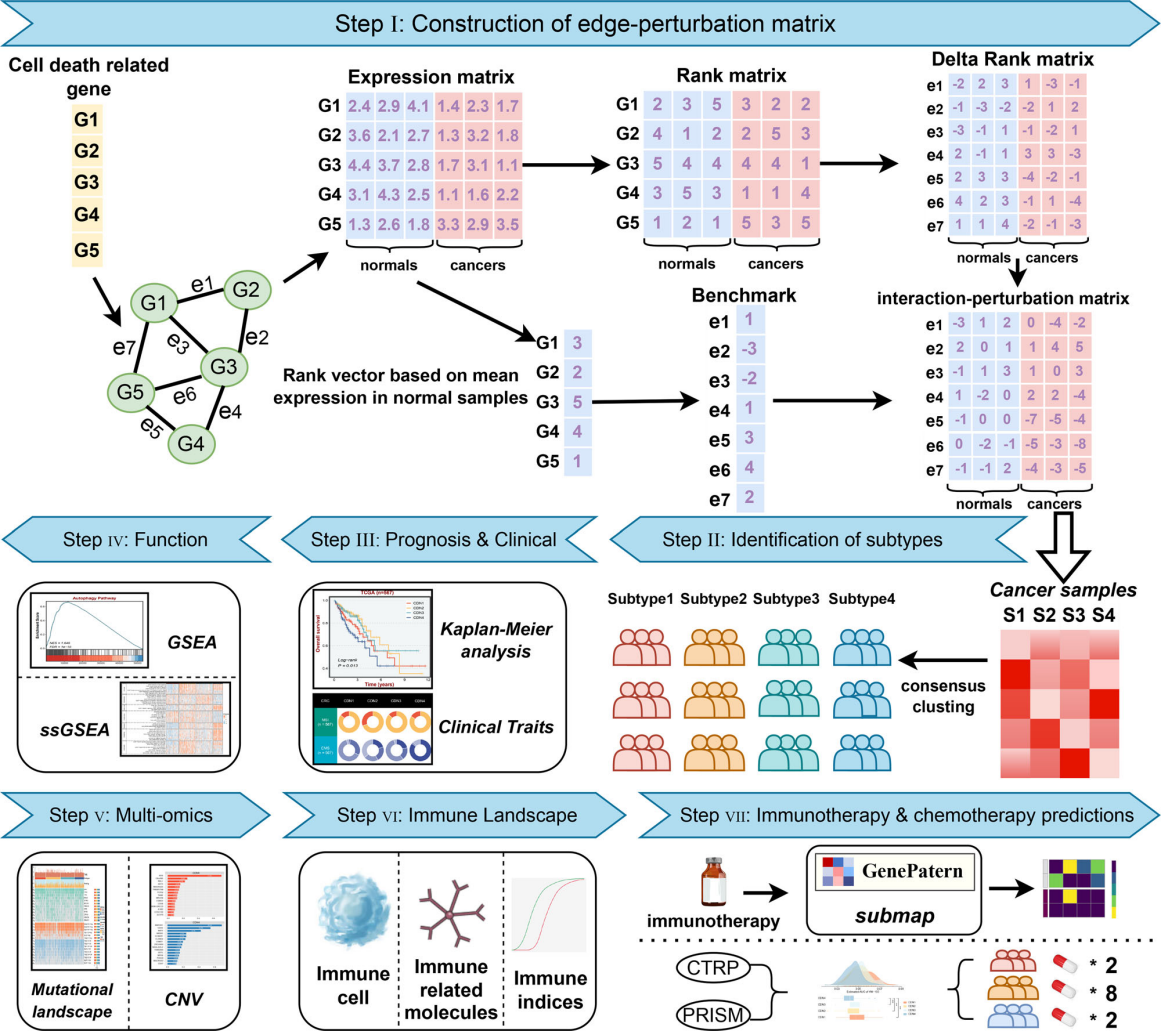
The overall flow of this study. The background network consists of five cell death related genes and five interactions. There were three normal samples (blue) and three cancer samples (pink). The genes were sorted according to the expression value of each sample to obtain a rank matrix. The rank matrix was converted into a delta rank matrix consisting of five rows and six columns representing the interactions and samples, respectively. The benchmark delta rank vector was calculated as the delta rank of the average expression value across all normal samples. The benchmark delta rank vector was subtracted from the rank matrix to obtain the interaction perturbation matrix. The interaction perturbation matrix was then used to cluster the colorectal cancer samples to reveal new network-based subtypes. The identified subtypes had different characteristics, including prognosis, phenotypic traits, multi-omics, immune infiltration, and prediction of treatment effects.

The analysis revealed that scores pertaining to cell death pathways were significantly elevated in colorectal cancer samples compared to normal tissues (Supplementary Fig. 1a, b). Dimension reduction analysis, conducted on the basis of cell death genes, indicated a minimal overlap between the colorectal cancer and normal samples. This suggests an enhanced activity of cell death pathways in colorectal cancer samples relative to normal tissues (Supplementary Fig. 2a, b). To generate the interaction-perturbation of all gene pairs from the cell death interplay network, we introduced a four-step pipeline as previously reported^10^ (Fig. 1). CRC tissues from TCGA-CRC (*n* = 567) and normal colorectal tissues from GTEx database (*n* = 304) were regarded as tumor and normal input, respectively. The initial rank matrix was obtained based on the rank of each gene in a single sample, which was subsequently converted to the delta rank matrix using subtraction in the same direction of gene interactions. Furthermore, gene interaction was relatively stable and conservative in normal tissues^12,18^, thus serving as the benchmark for calculating the interaction-perturbation matrix in tumor tissues. Collectively, we generated a perturbation network consisting of 8403 edges and 1056 nodes. Previous evidence has demonstrated that biological networks are commonly scale-free distribution^19^, which was in line with our constructed network (*R* = −0.963, *P* < 0.001; Supplementary Fig. 2c). Notably, relative to normal samples, tumor samples exhibited stronger perturbations (Supplementary Fig. 2d) and the range of interaction perturbation is also broader range, suggesting a higher variation (Supplementary Fig. 2e). These results indicated that the cell death interplay network was overall stable in normal samples, whereas it significantly perturbated in tumor samples.

### Perturbation of cell death networks deciphered four heterogenous subtypes

**Fig. 2:**
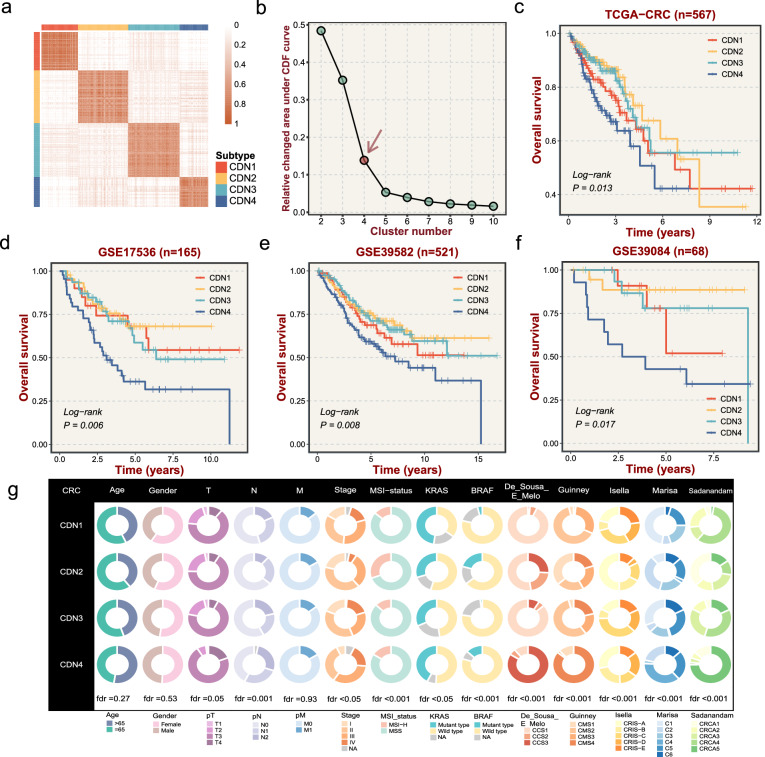
Calculation of interaction-perturbation in the cell death interplay network. **a** The consensus score matrix of all samples when k = 4. A higher consensus score between two samples indicates they are more likely to be grouped into the same cluster in different iterations. **b** For the relative change of area under the CDF curve, the point with insignificant increase is selected as the best K value. **c**–**f** Kaplan–Meier curves of overall survival with log-rank test for four CDN subtypes in TCGA and three validation datasets. **g** Correlations of four CDN subtypes with clinical characteristics and previous CRC classifications in the TCGA-CRC dataset. Gray areas represent missing value.

Previous studies have demonstrated that network perturbation could more reliably better characterize the biological state of bulk tissues^10–12^. Here, representative perturbation features that can strikingly distinguish normal and tumoral tissues as well as maintain significant heterogeneity across tumor samples were retained for subtype discovery, which formed a sub-network including 4203 edges and 924 nodes. Specifically, this sub-network also fitted a scale-free distribution (*R* = −0.977, *P* < 0.001, Supplementary Fig. 2f). Afterward, Consensus clustering was performed with different k (k = 2–9) clusters according to the perturbation matrix of representative features. Results of cumulative distribution function (CDF) curve and consensus score matrix suggested that the optimal division was achieved when k  = 4 (Fig. 2a, b and Supplementary Fig. 2g). Thus, four subtypes were decoded from cell death network (CDN1, *n* = 128, 22.6%; CDN2, *n* = 170, 30%; CDN3, *n* = 170, 30%, and CDN4, *n* = 99, 17.4%). Kaplan–Meier analysis displayed significant survival differences among four subtypes, with CDN2 having the best prognosis and CDN4 possessing the worst prognosis with a 5-year survival rate of about 50% (*P* = 0.013, Fig. 2c).

To further validate the reproducibility and stability of four CDN subtypes in independent cross-platform cohorts, we retrieved three internal datasets to perform multiclass predictions using the NTP algorithm. Initially, 799 subtype-specific genes were identified via differential expression analysis. Subsequently, cluster prediction was achieved by the NTP framework integrated 799 featured genes and three testing expression matrices. Encouragingly, the sample subtypes displayed similar transcriptome profiles across different cohorts (Supplementary Fig. 3a–c). In line with the prior findings, CDN4 presented dismal prognosis relative to other subtypes in all testing datasets (Fig. 2d–f). In addition to similar transcriptome and clinical traits, four subtypes also demonstrated analogical proportion (Supplementary Fig. 3d). Taken together, the above suggested that our CDN taxonomy was robust and reproductive in cross-platform cohorts.

### Correlations of four subtypes with clinical characteristics and classical subtypes

Subsequently, we compare the distribution of clinical characteristics among four subtypes (Fig. 2g). CDN1 was closely associated with *KRAS* mutation (chi-square test, fdr <0.05), whereas CDN2 was related to *BRAF* mutation (chi-square test, fdr <0.001) and high level of microsatellite instability (MSI-H) (chi-square test, fdr <0.001) (Supplementary Fig. 3f, g). In addition, CDN4 was related to advanced TNM stage (chi-square test, fdr <0.05), consistent with the poor prognosis of CDN4.

To further compare our CDN taxonomy with previously developed CRC classifications, patients were reclassified according to criteria based on previous studies from Guinney et al.^5^, Isella et al.^6^, De Sousa E Melo et al.^7^, Sadanandam et al.^8^, and Marisa et al.^9^. Intriguingly, the results revealed strong associations between four CDN subtypes and previous classifications, indicating the molecular convergence in CRC (Fig. 2g). Specifically, CDN1 was linked to the canonical CMS2, CRCA4, CCS1, CRIS-C/CRIS-D, and CIT1; CDN2 was associated with CMS1, CCS2, CRCA1, CRIS-A, and CIT2; CDN3 was enriched in CMS2, CCS1, CRCA3, CRIS-C, and CIT5. In contrast, CDN4 was related to more aggressive classification features, including CMS4, CCS3, CRCA5, CRIS-B, and CIT4. Of note, only a fraction (20%) of our signature genes overlapped with signature genes of previous CRC classifications, suggesting that our classification has specific biological characteristics as well as more room for exploration (Supplementary Fig. 3e).

### Biological characteristics of four subtypes

**Fig. 3:**
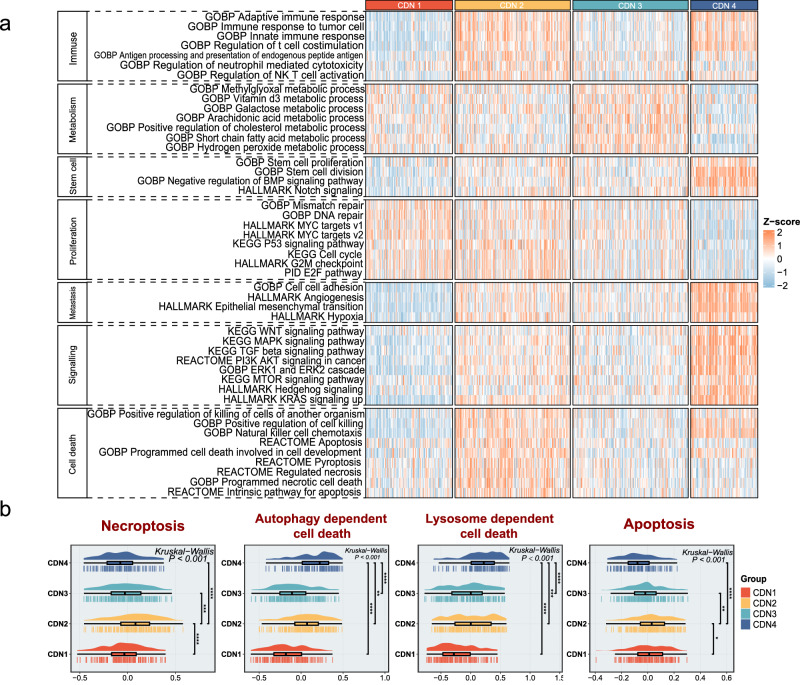
Biological processes associated with the CDN subtypes. **a** Heatmaps of biological processes for four CDN subtypes in TCGA-CRC datasets. High and low ssGESA scores are represented in red and blue, respectively. **b** The degree of enrichment of the four CDN isoforms in four different cell death pathways (necroptosis, autophagy-dependent cell death, lysosomal-dependent cell death, and apoptosis). ns fdr > 0.05, *fdr < 0.05, **fdr < 0.01, ***fdr < 0.001, ****fdr < 0.0001.

To characterize the biological behaviors in four subtypes, we calculated the variation of biological pathways via the GSVA algorithm. The results indicated that biological functions differed dramatically among four subtypes (Fig. 3a). Specifically, CDN1 was endowed with moderate metabolic activity and low immune activity, which demonstrated significant enrichment in proliferation pathways, such as cell cycle, G2M checkpoint, and MYC targets. CDN2 was also enriched in proliferation pathways but displayed conspicuous enrichment in immune-activated and multiple cell death pathways. The metabolic pathways, such as lipid, vitamin, and galactose metabolism, were particularly evident in CDN3. CDN4 was associated with significant aggressive features, including cancer stem cell-related pathways, metastasis-related pathways, and multiple cancer-related signaling pathways. Subsequently, the enrichment of the four subtypes in 11 cell death pathways was explored (Fig. 3b and Supplementary Fig. 4a–g), and we observed that CDN2 exhibits high levels in most of the cell death pathways, especially in necroptosis (Kruskal–Wallis test, *p* < 0.001) and apoptosis (Kruskal–Wallis test, *p* < 0.001). While CDN1 in most of the cell death pathways showed a lower level, CDN3 displayed moderate levels in all cell death-related pathways. CDN4 was mainly enriched in autophagy (Kruskal–Wallis test, *p* < 0.001) and lysosome-dependent cell death pathways (Kruskal–Wallis test, *p* < 0.001). Overall, CDN1 was defined as a proliferative subtype because of the significant proliferative activity. CDN2 was defined as an immune subtype with strong cell death activity. CDN3 had high metabolic activity as metabolic activity. As a result of high cell stemness as well as high levels of invasive tumor pathways, CDN4 was defined as an aggressive subtype, along with high autophagic activity.

### CDNs heterogeneity under single cells

In this study, we have successfully identified CDNs-like epithelial cells from tumor samples based on characteristic gene markers. Each subtype of CDNs-like epithelial cells, when analyzed using Uniform Manifold Approximation and Projection (UMAP) for dimensional reduction, exhibited a distinct distribution pattern (Supplementary Fig. 5a). This pattern suggests the presence of transcriptional heterogeneity among the four CDNs-like epithelial cells. Intriguingly, this heterogeneity is not only observed across different samples but is also evident within individual samples (Supplementary Fig. 5b). Noteworthy, CDN4-like cells showed the highest scoring in autophagy pathways (Kruskal–Wallis test, *p* < 0.001, Supplementary Fig. 5c), which is consistent with the bulk data analysis.

### Multi-omics landscape of four subtypes

**Fig. 4:**
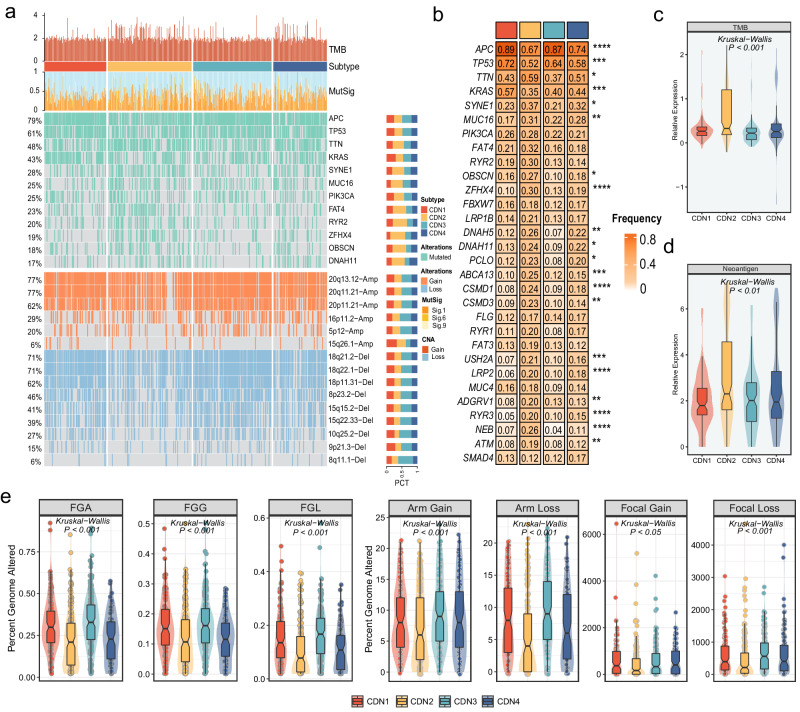
Multi-omics landscape of the CDN subtypes. **a** Landscape of frequently mutated genes and CNV of amplification and deletion among the CDN subtypes. **b** Analysis of mutational differences in 30 frequently mutated genes (FMGs) among the four CDN subtypes. **c**, **d** Distributions of TMB (**b**) and Neoantigen (**c**) among the CDN subtypes. **e** Comparison of FGA, FGG, FGL, arm gain, arm loss, focal gain, and focal loss among the four CDN subtypes. FGA fraction of genome alteration, FGG fraction of genome gained, FGL fraction of genome lost, *fdr < 0.05, **fdr < 0.01, ***fdr < 0.001, ****fdr < 0.0001.

To characterize the molecular profiles of four CDN subtypes at the mutational and CNV levels. First, we depicted the mutational landscape of four subtypes (Fig. 4a), which showed that CDN1 and CDN3 were prominent in *APC*, *TP53*, and *KRAS* mutations, while *TTN*, *SYNE1*, and *MUC16* performed higher mutation frequencies in CDN2 and CDN4 (Fig. 4b). Notably, *KRAS* mutations can stimulate tumor cell proliferation and growth, which leads to a poor prognosis, while mutations are less abundant in CDN2. Overall, most driver genes in CDN2 have the highest mutation frequency, and tumor mutation burden (TMB) is also the highest of the four subtypes (Kruskal–Wallis test, *p* < 0.001, Fig. 4a, c). Higher TMB tend to generate more tumor neoantigens (Kruskal–Wallis test, *p* < 0.01, Fig. 4d), which are more likely to stimulate immune activation. In addition, the burden of CNV (gain or loss) at the level of bases, fragments, and chromosome arms was highest in CDN3 (Fig. 4e). That said, CDN3 was inclined to be CNV-driven, whereas CDN2 was inclined to be mutation-driven.

### The immune landscape of four subtypes

**Fig. 5:**
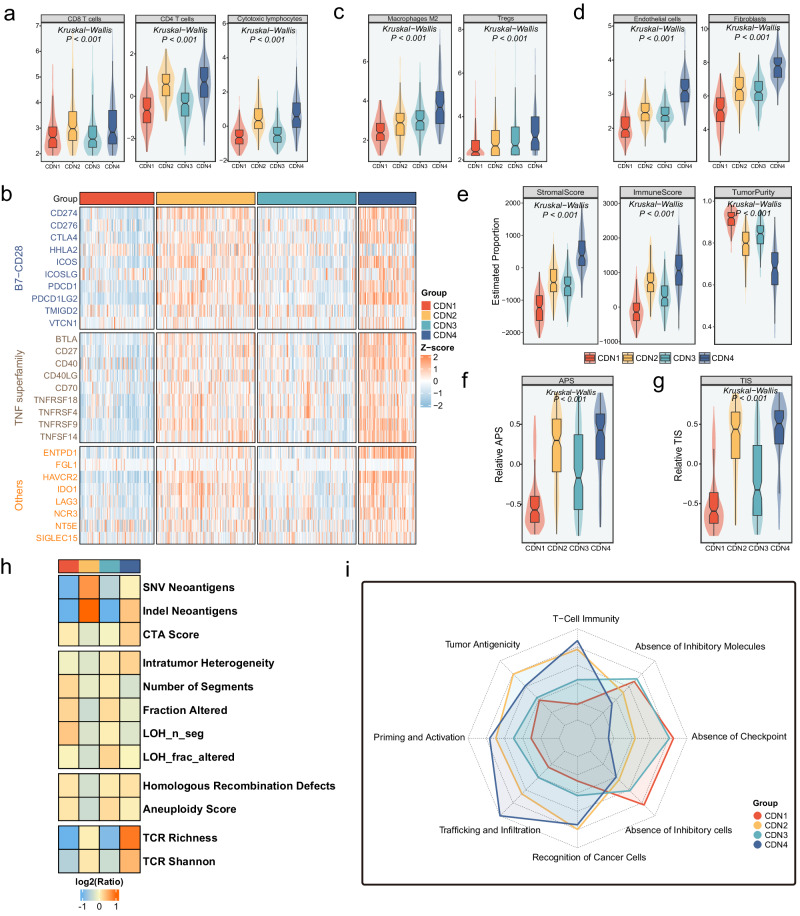
Immune landscape of four CDN subtypes. **a** The infiltration difference of CD8 T cells, CD4 T cells, and cytotoxic lymphocytes among four CDN subtypes. **b** Assessment of infiltration abundance of 27 immune checkpoints among four CDN subtypes. **c** The infiltration difference of macrophages M2, and tregs among four CDN subtypes. **d** The infiltration difference of endothelial cells, and fibroblasts among four CDN subtypes. **e** Comparison of stromal score, immune score and tumor purity among four clusters. Comparison of APS (**f**) and TIS (**g**) among four clusters. **h** Comparison of 12 immunogenicity associated indicators among the four CDN subtypes, the cell represented by the mean value of corresponding cluster divided by the overall mean value. **i**. Radar plots showed the immunogram patterns of CDN subtypes.

Previous studies have demonstrated that the composition of immune cells within infiltrating CRC tumors is heterogeneous, and the key players are continuously subject to microenvironmental changes. Thus, to further explore the immune landscape of four subtypes, the ssGSEA was employed to measure the abundance infiltration of 28 immune cells. The results displayed that most immune cells infiltrated mainly in CDN2 and CDN4, with CDN3 as an intermediate location, while CDN1 had the lowest level of immune cell infiltration (Supplementary Fig. 6a). Especially, activated effector cells that serve an instrumental role in antitumor immunity, such as activated CD8 T cells (Kruskal–Wallis test, *p* < 0.001), activated CD4 T cells (Kruskal–Wallis test, *p* < 0.001), and cytotoxic lymphocytes (Kruskal–Wallis test, *p* < 0.001, Fig. 5a), were highly infiltrated in CDN2 and CDN4. In addition, immune checkpoints such as B7-CD28 family member proteins and TNF superfamily were highly expressed in CDN2 and CDN4 (Fig. 5b). Among them, the immune checkpoint CD40L binding to CD40 could increase the immunomodulatory ability of DCs, induce effector cell proliferation and enhance immunotoxicity to tumor cells, which further indicated that CDN2 and CDN4 had strong immune activation ability. However, although CDN4 had a higher immune score, macrophages M2, regulatory T cells, and stromal cells were highly infiltrated in CDN4 with a higher stromal score, which demonstrated that CDN4 was an immune hot but suppressed tumor microenvironment. (Fig. 5c–e). Furthermore, the APS and TIS score also demonstrated that CDN2 and CDN4 had stronger antigen presentation capacity and better response to immunotherapy (Kruskal–Wallis test, *p* < 0.001, Fig. 5f, g). CDN1 possesses highest tumor purity accompanied by low levels of immune infiltration (Kruskal–Wallis test, *p* < 0.001, Fig. 5e). Moreover, CDN2 had higher levels of SNV neoantigen, indel neoantigen, and CDN4 was featured by higher TCR Richness, TCR Shannon, homologous recombination deficiency, intratumoral heterogeneity, and cancer-testis (CTA) scores (Fig. 5h). Taken together, CDN1 was defined as the immune desert subtype because of insufficient immune cell infiltration. CDN2 had high immune activity and was defined as the immune activating subtype. CDN3 is the intermediate immune subtype. With the strong immune activation but suppressed microenvironment, CDN4 was defined as the immune suppressive subtype.

### Immunotherapy

To systematically assess the immunotherapeutic potential of four CDN subtypes, we need to establish a comprehensive assessment of tumor immunity. Karasaki et al. proposed an immunogram for quantifying the cancer-immunity cycle^20^. Hence, we applied the immunogram to assess the potential for subtypes to benefit from immunotherapy (Fig. 5i). CDN1 and CDN3 exhibited low immune cycle scores, in line with previous findings indicating inadequate immune infiltration. CDN2 and CDN4 displayed higher levels of immune activation and antitumor related immune cycle scores, along with relatively high immune checkpoint expression. Thus, CDN2 and CDN4 are more likely to benefit from immunotherapy. In addition, we applied an investigational 18-gene signature termed TIS, which can be used to measure a pre-existing but suppressed adaptive immune response within tumors. As shown in Fig. 5g, CDN2 and CDN4 displayed higher levels of TIS scores. SubMap analysis also revealed significant expression similarity between CDN2 and responders in CAR-T, anti-PD-1, and anti-CTLA-4 treatment cohorts (Supplementary Fig. 6b). Moreover, CDN2 and CDN4 might be sensitive to anti-PD-1/CTLA-4 combination immunotherapy (Supplementary Fig. 6c). These results demonstrated that CDN2 might benefit from immunotherapy, while CDN4 with the poorest prognosis might benefit from multi-targeted immunotherapy. Overall, results indicated that CDN2 and CDN4 might derive clinical benefit from immunotherapy, whereas CDN1 and CDN3 may not fit it due to the high cost and underlying immune-related adverse events that accompany low response potential.

### AOC3: a predictive molecule of CDN4 indicate poor prognosis

**Fig. 6:**
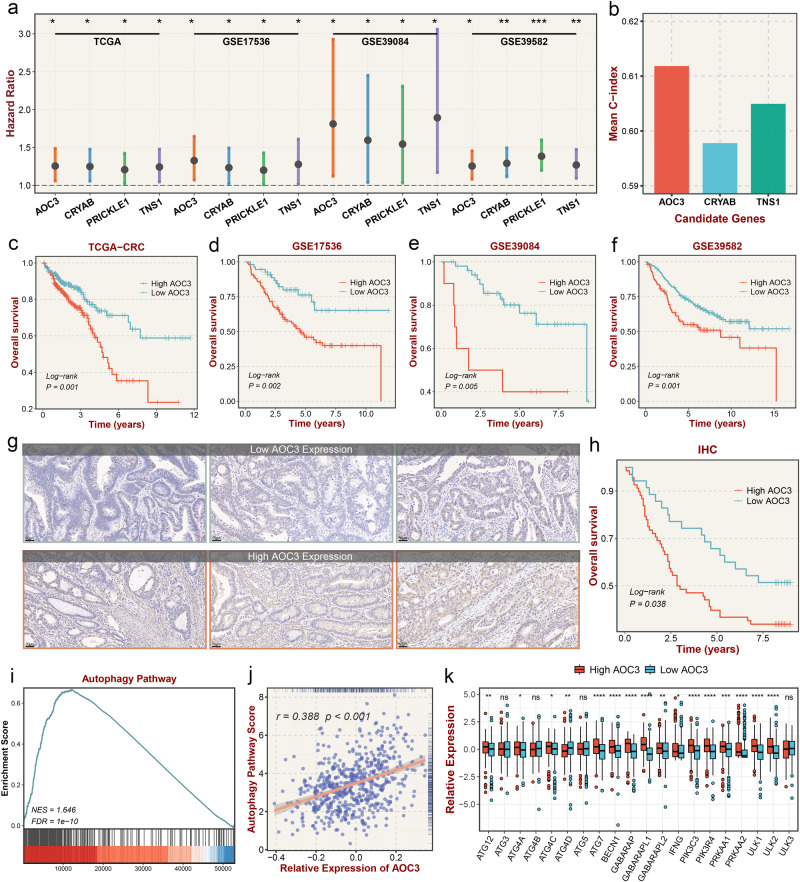
The specific molecular of the CDN4 subtype and immunohistochemical staining. **a** Univariate Cox analysis of CDN4 feature genes with *p* < 0.05 and HR > 1 in TCGA and GEO prognosis cohorts. **b** Mean C-index of the candidate genes. **c**–**f** Kaplan–Meier curves of overall survival with the log-rank test between the high *AOC3* group and low *AOC3* group in TCGA and three validation datasets. **g** Representative immunohistochemistry (IHC) staining images of CRC tissue microarrays in low *AOC3* tumor expression and high *AOC3* tumor expression tissues. **h** Kaplan–Meier curves of overall survival with the log-rank test between the high *AOC3* H-scores group and low *AOC3* H-scores group in IHC arrays. **i** The molecules significantly associated with *AOC3* were enriched in regulation of autophagy pathway. **j** Correlation analysis of *AOC3* with regulation of autophagy pathways score. **k** Differential expression of autophagy genes in different *AOC3* expressions. ns fdr>0.05, *fdr < 0.05, **fdr < 0.01, ***fdr < 0.001, ****fdr < 0.0001.

To further explore the CDN4 subtype with the worst prognosis, a total of 30 CDN4 feature genes kept AUC > 0.9 in four independent prognostic cohorts. The result of univariate Cox analysis based on CDN4 signature genes demonstrated that *AOC3*, *CRYAB*, *PRICKLE1*, and *TNS1* were risk factors with prognostic significance and showed significant consistency in the four cohorts (Fig. 6a). However, only three genes were eligible for both conditions, and the mean C-index of *AOC3* (mean C-index: 0.612, Fig. 6b) was the highest compared with *CRYAB* (mean C-index: 0.598) and *TNS1* (mean C-index: 0.605). In cohorts with overall survival information from TCGA and GEO, the low *AOC3* group performed a significantly better prognosis than the high *AOC3* group (Fig. 6c–f). To further validate the prognostic potential of *AOC3*, we divided the samples into the high H-scores group and the low H-scores group based on the H-scores of *AOC3* from 103 successfully stained cancer samples in human tissue microarrays (Fig. 6g). The survival analysis result was consistent with previous analyses that the high *AOC3* group had a significantly worse prognosis than the low *AOC3* group (Fig. 6h). GSEA analysis showed that the molecules significantly associated with *AOC3* were mainly enriched in the regulation of autophagy pathway, and *AOC3* showed a significant association with the regulation of autophagy (*R* = 0.388, *P* < 0.001, Fig. 6i–j). In addition, high expression of *AOC3* is often accompanied by high expression of autophagy genes (Fig. 6k).

## Discussion

As developing studies focus on cell death, the critical role of cell death pathways in tumor development and progression has been gradually revealed. Previous research indicated that imbalanced or defective cell death signaling might be essential in CRC evolution and poor response to chemotherapy and radiotherapy^21^. In contrast, other studies demonstrated that induction of ferroptosis could reverse drug resistance to inhibit tumor growth^22^. Moreover, clinical trials have demonstrated that pharmacological and molecular blockades of autophagy could improve anticancer therapy efficacy^23^. However, different cell death pathways tend to interact in tumors compared to operating alone. For example, apoptosis and autophagy could act synergistically to inhibit the signaling of other pathways to kill tumor cells^24^. Furthermore, the potential molecular mechanisms of the different cell death pathways show considerable overlap, which sets a foundation for studying crosstalk among different cell death pathways. To explore various cell death pathways integratively, we constructed an interaction network by retrieving critical molecules associated with 11 cell death pathways from previously reported. As we all know, biological networks remain relatively constant across time and conditions, which can better characterize the biological state of the overall tissue^10–12^. At the same time, this biological network generally remains stable in normal tissues and highly variable in tumor tissues^18^, which was also proved in this study. Therefore, based on this relatively stable cell death network, we identified four heterogeneous CDN isoforms and characterized their biological properties, immunological traits, and genomic features of four subtypes. With the validation of multiple cross-platform cohorts, our constructed classification method proved stable and reproducible. Notably, although the CDN subtype was significantly associated with the published classification for CRC, there were limited overlapping signature genes between classical classifications and CDN, indicating a significant molecular convergence but having distinct characteristics.

According to biological function, CDN1 was defined as the proliferation-promoting isoform and CDN3 was defined as the metabolic isoform. Compared with CDN3, CDN1 has a relatively poor prognosis, possibly due to the combined effects of significant proliferation and enriched *KRAS* mutations^25,26^. Despite the high tumor purity, CDN1 showed a neutral malignancy from prognosis analysis, which was consistent with previous reports^27^. Coincidentally, both CDN1 and CDN3 showed low TMB and immune desert TME and were predicted to respond poorly to immunotherapy. Additionally, CDN3 has higher copy number deletions and amplifications at the level of bases, segments, and chromosome arms. As previously reported, high load of copy number promotes immune escape, leading to poor immunotherapy^28^. And CDN3 showed elevated lipid metabolism and glucose metabolism. Abnormal metabolic activities promote the growth and proliferation of cancer cells, which is currently considered a hallmark in numerous malignant tumors, including CRC^29^, meaning these individuals may benefit from antimetabolite medication.

With the highest TMB, the CDN2, considered a mutation-driven subtype, presents the highest mutation frequency in most genes. Tumor cells with high TMB generally present high levels of neoantigens, which can induce cytotoxic T lymphocyte activation and proliferation to eradicate tumor cells^30^. In CDN2, some cell death pathways such as apoptosis, ferroptosis, necroptosis, and pyroptosis also play a synergistic role in antitumor immunity. According to previous reports, necroptotic cells can release DAMP to DC cells, triggering antigens, and thereby activating cytotoxic CD8 + T lymphocytes^31^. Pyroptosis inhibits Tregs differentiation and macrophage death to promote adaptive responses^32^. Correspondingly, CDN2 showed abundant infiltration of various immune cells and high immunogenicity in immunology analysis. Additionally, MSI-H tumors, which are widely reported to be linked to a better immunotherapy response, are also enriched in CDN2^33^. As the microsatellite instability subtype from classical subtypes, we observed higher proportions of CCS2 and CMS1 in CDN2. Taken together, these results suggest that CDN2 has great potential to benefit from immunotherapy, which has also been validated in several immunotherapy cohorts.

The CDN4 subtype characterized by mesenchymal transcription has a poor prognosis, matching CMS4, CRIS-B, CCS3, and CRCA5. Downregulation of BMP signaling pathways and upregulation of NOTCH and WNT signaling pathways can enhance stem cell activation^34^, while low tumor purity and high enrichment in cancer stemness signatures also suggest a malignant phenotype of CDN4. Consistent with previous studies, autophagy related pathways significantly enriched in CDN4 dominated by advanced tumors than other subtypes, which was validated at the single-cell level. And autophagy in established solid tumors can help in response to intracellular and environmental stresses such as nutrient shortages, hypoxia, cytotoxicity, or cancer treatments, which favor tumor progression^35^. Autophagy stimulation can also lead to the degradation of EMT inducers, thereby accelerating tumor invasion and metastasis^36^. In terms of antitumor immune responses, the immune evasion mechanism of CDN4 is mainly due to a richer infiltration of immunosuppressive cells and immunosuppressive molecules in the tumor microenvironment, such as MDSCs and M2 macrophages. In addition, autophagy promotes immunity by degrading MHC-I escape, leading to intrinsic resistance to tumor immunotherapy^37^. We found that CDN4 only responded in combined immunotherapy with anti-PD-1/CTLA-4, which may contribute to the dual blockade of PD-1/CTLA-4 increasing effector T-cell activity to enhance tumor suppression^38^. For CDN4, which has a higher degree of malignancy, we identified *AOC3* as its unique biomarker. *AOC3* possessed superior CDN4 predictive performance and significant prognostic significance, and has been shown to be an independent prognostic risk factor in previous CRC clinical studies^39^. As an endothelial adhesion molecule, *AOC3* has previously been implicated to play a role in lymphocyte-endothelial interactions to promote tumor growth. Our study also showed that *AOC3*-related genes were mainly enriched in the autophagy pathway and showed significant correlation. Thus, autophagy may be another mechanism linking *AOC3* and cancer progression. Recent studies have shown that autophagy can be used as a diagnostic indicator for postoperative patients, and the expression of autophagy-related proteins such as Beclin1 in tumor tissues is related to tumor metastasis and recurrence^40^. Therefore, *AOC3* is promising to be used to identify CDN4 with autophagic properties, which may benefit from autophagy-targeting therapeutic regimens.

In general, based on the construction of the cell death crosstalk network, which is more stable and effective than gene features, our study established four stable CRC molecular subtypes that could predict prognosis and guided treatment. The study of tumor heterogeneity in a relatively stable background network facilitates the rational assignment of individuals with tumors, which may contribute to precise clinical management. In addition, our study also identified *AOC3* as a potential biomarker for identifying individuals with poor prognosis associated with autophagic properties, which may facilitate clinical prognostic evaluation and personalized management in CRC. The study also has certain shortcomings, as the analysis of the results is limited by the retrospective of the study, such as possible confounding factors of imbalance between subtypes and non-randomized patient treatment selection. Accordingly, more prospective and basic clinical trials are needed to validate the conclusions further.

## Method

### Data collection and preprocessing

Four independent CRC cohorts were retrieved from The Cancer Genome Atlas (TCGA, https://portal.gdc.cancer.gov) and Gene Expression Omnibus (GEO, http://www.ncbi.nlm.nih.gov/geo) databases, including TCGA-CRC (*n* = 567), GSE39582 (*n* = 521), GSE39084 (*n* = 68), and GSE17536 (*n* = 165). The criteria for our screening cohort were as follows: (1) primary tumor tissue samples; (2) RNA expression data available; (3) no preoperative chemotherapy or radiotherapy; (4) survival information available and survival time >30 days. The detailed baseline was summarized in Supplementary Table 1. Somatic mutation and copy number variation (CNV) in TCGA-CRC were also downloaded from the TCGA portal. RNA-seq count data of TCGA-CRC were converted to transcripts per kilobase million (TPM) and further log-2 transformed. RNA-seq data (log_2_TPM) of 308 normal colorectal tissues were obtained from Genotype-Tissue Expression (GTEx, http://gtexportal.org). Raw data of CRC microarrays from the GPL570 platform were processed and normalized using the robust multichip analysis (RMA) algorithm implemented in the *Affy* package. Furthermore, five immunotherapy cohorts (Nathanson cohort, GSE126044, GSE115821, GSE100797, and GSE91061) with expression profiles and immunotherapy information were also included in this study.

### Interplay network construction of cell death pathways

We comprehensively retrieved cell death-related genes from previous literature and Molecular Signature Database (MSigDB, https://www.gsea-msigdb.org/gsea/msigdb). In total, 1339 genes of 11 cell death pathways were collected (Supplementary Data 1). This study aimed to investigate molecular heterogeneity from both nodes and interactions of different gene sets. A pathway-based analysis requires a functional network of protein interactions predicted by candidate genes^10^. Thus, these genes were subjected to the Search Tool for the Retrieval of Interacting Genes (STRING, https://string-db.org/)^41^, which further generated an interplay network of 11 cell death pathways in the form of protein-protein interactions (PPI). Gene pairs with confidence >0.8 were retained for background construction. Ultimately, an interplay network consisting of 1056 nodes and 8403 interactions was constructed (Supplementary Data 2).

### Calculation of interaction-perturbation in the cell death interplay network

As previously reported, biological networks remain stable and conservative in normal tissues, whereas are significantly perturbated in tumor tissues^12,18^. In this study, CRC tissues from TCGA-CRC (Illumina HiSeq 2000) were regarded as tumor inputs. Nevertheless, TCGA database only includes tumor-adjacent tissues, which were also dramatically perturbated in biological networks relative to normal tissues. Thus, colorectal tissues from GTEx (Illumina HiSeq 2000) were regarded as normal input, which are derived from healthy donors. Figure 1 illustrates the interaction-perturbation construction of a small network example composed of five genes and five interactions. In brief, this pipeline mainly encompassed four steps:

1. In tumor and normal expression matrix, the rank of each gene in a single sample was calculated based on the expression value. Here, we generated the rank matrix.
2. According to gene interactions within the background network, we utilized subtraction in the same direction of gene interactions to convert the gene rank matrix into the delta rank matrix.
3. Previous evidence has demonstrated that biological networks are stable and conservative in normal tissues but perturbated in tumor tissues. Hence, the average delta rank of each gene pair in normal tissues was defined as a benchmark of each gene pair in tumor tissues.
4. Subsequently, the delta rank matrix minus the corresponding benchmarks of all gene pairs to form the interaction-perturbation matrix, which was used for next analysis (See details in the Supplementary Method).

### Consensus clustering

The interaction-perturbation matrix was used to identify heterogeneous CRC subtypes via the consensus clustering algorithm implemented in the *ConsensusClusterPlus* package^42^. The clustering features were determined based on the following criteria: (1) significantly distinguished tumor samples from normal samples; (2) maintained heterogeneity among all tumor samples. As previously reported, the top 6000 interactions with significant differences between normal and tumor samples as well as the top 6000 with high standard deviation (SD) in tumor samples are intersected to form 4203 features for consensus clustering. All derived partitions of cluster K (2-10) were summarized by clustering co-classification matrices. The program performed 1000 iterations on resample rate of 0.8 by employing the partitioning around medoids approach and 1-Spearman correlation distance. Furthermore, the optimal cluster was determined synthetically using the consensus score matrix and cumulative distribution function (CDF) curve.

### Investigating the stability of molecular signatures

Nearest template prediction (NTP) is a single-sample-based flexible class prediction method that enables cross-platform and multiclass predictions with confidence assessment^43^. Signature genes of each subtype were determined as previously reported (Supplementary Data 3)^44^. In testing datasets, NTP was applied to identify four defined subtypes using the expression profiles of signature genes. Samples with FDR < 0.2 were considered successfully classified. Furthermore, this study incorporated a CRC single-cell cohort (GSE178341), specifically focusing on the extraction of previously annotated tumor epithelial cells. To categorize these cells into groups reflective of the various subtype transcriptomes, we used single-sample Gene Set Enrichment Analysis (ssGSEA) scores to identify epithelial cell groups based on signature genes unique to each subtype^45^.

### Functional analysis

To explore the underlying biological function of each subtype, we performed gene set enrichment analysis (GSEA)^46^, and terms with FDR < 0.05 were considered significant. In addition, a total of 9997 gene sets were retrieved from MSigDB, and the gene expression matrix was converted into a pathway enrichment scoring matrix through gene set variation analysis (GSVA)^47^ algorithm. Characterized pathways within the four subtypes were further revealed using the *limma* package, with thresholds of fdr <0.05 and |log2FC | >0.2.

### Immune landscape

The ssGSEA^48^ was employed to measure the abundance infiltration of 28 immune cells in bulk tumor samples. Additionally, 27 key immune checkpoints, including the B7-CD28 family^49^, TNF superfamily^50^, and other molecules^51,52^, were collected. Immunogenic indicators were also retrieved for investigating the underlying immune escape mechanisms of four subtypes (Supplementary Table 2). Cancer-immunity cycle (CIC) proposed by Karasaki and colleagues^20^ consists of eight axes of the immunogram score (IGS), which was measured using ssGSEA algorithm.

### Multi-omics landscape

Frequently mutated genes (FMGs) were genes with the top 30 mutation frequency. GISTIC 2.0^53^ was applied to identify genomic regions with significant amplifications and deletions, and we quantified the overall genomic changes by calculating genomic alterations (FGA) fractions, fractions of genomes lost (FGL), and fractions of genomes obtained (FGG) to quantify overall genomic changes. Additionally, CNV burdens at the arm and focal level were quantified based on areas of recurrent alteration originating from the GISTIC 2.0 pipeline. Tumor mutational burden (TMB) was defined as the number of somatic, coding, base substitution, and indel mutations per megabase of genome examined by using nonsynonymous and frameshift indels at 5% limit of detection^54^. TMB was calculated using single nucleotide variant (SNV) data from the TCGA database with whole-exome sequencing (WES) profiling.

### Immunotherapeutic assessment

To evaluate the therapeutic potential of immunotherapy in each subtype, four cohorts with both transcriptome data and immunotherapeutic information were enrolled. Following the Response Evaluation Criteria in Solid Tumors (RECIST v1.1) criteria, responders and nonresponders were defined as complete/partial response (CR/PR) and stable/progression disease (SD/PD), respectively. Subclass Mapping (SubMap) could quantify the expression similarity between different subgroups in two datasets^55^. In this study, we utilized the SubMap algorithm to calculate the molecular similarity between responders/nonresponders and four subtypes, further evaluating the therapeutic potential of immunotherapy in each subtype. Moreover, T-cell inflammatory signature (TIS)^56^ is an investigational 18-gene signature that measures a pre-existing but suppressed adaptive immune response within tumors, which was quantified using the ssGSEA method. A high TIS suggests a higher sensitivity to immunotherapy.

### The specific molecular of the CDN4 subtype

To facilitate the identification of CDN4 phenotypes in CRC samples, feature genes of the CDN4 subtype with *P* < 0.05 and HR > 1 by univariate Cox regression analysis were retained. The *pROC* package was applied to calculate the area under the receiver operating characteristic curve (AUC) to evaluate the CDN4-predicted power of feature genes, of which AUC > 0.9 were included (Supplementary Table 3). The performance of each candidate gene in predicting prognosis was comprehensively evaluated in all cohorts by C-index.

### Immunohistochemical staining and quantitative assessment

The CRC human tissue microarrays (HColA180Su16) were purchased from Shanghai Outdo Biotech Company (Shanghai, China), and the accordance clinical information, including 104 cancer and 76 adjacent non-cancerous tissues, were obtained from the company website. Immunohistochemistry (IHC) was performed using *AOC3* (1:600; Cat no. 66834-1-Ig, Proteintech, Wuhan, China). Each specimen was scored in term of staining intensity (no staining = 0, weak staining = 1, moderate staining = 2, and strong staining = 3). Multiplying the intensity score by the proportion of tumor cells staining generated H-scores.

### Statistics and reproducibility

All data processing, visualization, and statistical analysis were conducted in the R 4.2.1 software. Correlations between two continuous variables were assessed via Spearman’s correlation coefficient. Initially, we performed normality tests on these data sets. For data exhibiting normal distribution and homogeneity of variances, we employed the student t-test and one-way ANOVA to compare differences between two or multiple groups, respectively. In contrast, for data that are either non-normally distributed or do not meet the criteria for homogeneity of variances, comparisons between two groups or within multiple groups were conducted using the Wilcox test and Kruskal–Wallis test, respectively. Categorical variables were analyzed using the chi-square test. The *p*-values were adjusted using False Discovery Rate (FDR), particularly when performing multiple pairwise comparisons among groups. To verify the results, we conducted the experiments in triplicate, confirming their reproducibility.

### Reporting summary

Further information on research design is available in the Nature Portfolio Reporting Summary linked to this article.

### Supplementary information

Supplementary Information

Description of Additional Supplementary Files

Supplementary Data 1

Supplementary Data 2

Supplementary Data 3

Reporting Summary

## Data Availability

Public data used in this work can be acquired from the TCGA (https://portal.gdc.cancer.gov/) and GEO. Other data supporting the findings of this study are available from the corresponding author upon reasonable request.
