# Supplementary material for: Crosstalk of cell death pathways unveils an autophagy-related gene AOC3 as a critical prognostic marker in colorectal cancer
Source: Commun Biol. 2024 Mar 9;7:296. doi: 10.1038/s42003-024-05980-6 (PMC10924944; doi:10.1038/s42003-024-05980-6)
Supplement: Supplementary file 1 — Supplementary Information [file 42003_2024_5980_MOESM1_ESM.pdf]

Supplementary Methods

Removal of biased samples

The principal component analysis method was used to downscale the GTEx (n =308) and TCGA-CRC (n =567) data to observe the clustering trends of normal and tumor samples (**Supplementary Figure 1a**). The results showed that four of the normal samples had large deviation values, which may cause errors in the subsequent analysis. Therefore, we deleted these four samples. The results of the principal component analysis of the remaining normal (n =304) and tumor samples (n =567) showed stable and highly discriminatory clustering results (**Supplementary Figure 1b**).

Calculation of interaction-perturbation in the cell death interplay network

First, the gene expression matrix was sorted according to the level of gene expression values (the smallest and largest expression values correspond to the smallest and largest ranks, respectively), thereby converting it into a rank matrix. Subsequently, the delta rank matrix with rows and columns representing the interactions in the background network and samples was generated from the rank matrix. The $R_{x,s}$ represents the rank of gene G_x in sample s. Calculate the delta rank (represented by $\delta_{e,s}$) by subtracting the rank of each interconnected gene pair in the background network.

$$\boldsymbol{\delta_{e,s} = R_{x,s} - R_{y,s}}$$

Where genes G_x and G_y are connected by interaction e in the background network.

Gene interactions in normal samples are highly stable with little interaction perturbation. Hence, we assumed that the background network is very stable across all normal samples, and then use the interactions within the normal samples as the baseline network. We ranked the genes in the normal samples according to the mean gene expression and used the delta rank as the benchmark delta rank vector with the element represented by:

$$\boldsymbol{\delta_e}$$

where e is an interaction in the background network. This vector measures the average relative rank of gene pairs across all normal samples.

Each sample should be compared with the benchmark delta rank vector and the corresponding difference represents the gene interaction perturbation on the sample. The benchmark rank vector was subtracted from the rank of each sample to obtain the interaction-perturbation matrix with element $\Delta_{\boldsymbol{e},\boldsymbol{s}}$.

$$\Delta_{\boldsymbol{e},\boldsymbol{s}} = \boldsymbol{\delta}_{\boldsymbol{e},\boldsymbol{s}} - \boldsymbol{\delta}_{\boldsymbol{e}}$$

The interaction-perturbation matrix will be converted into a cancer sample matrix for subsequent clustering analysis.

Supplementary Figures

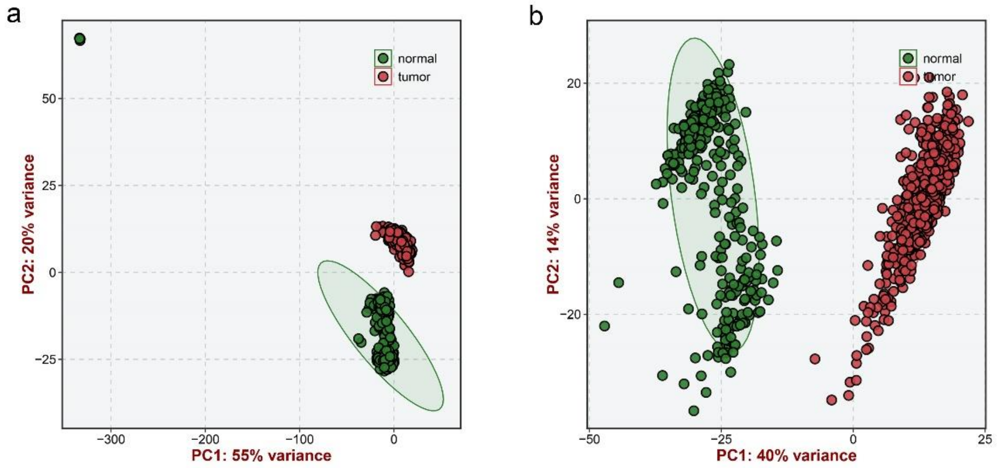

Supplementary Figure 1. Principal components analysis (PCA) of tumor and normal samples. a PCA analysis based on cell death-related genes in whole normal and tumor samples. **b** PCA analysis based on cell death-related genes after removing outlying samples.

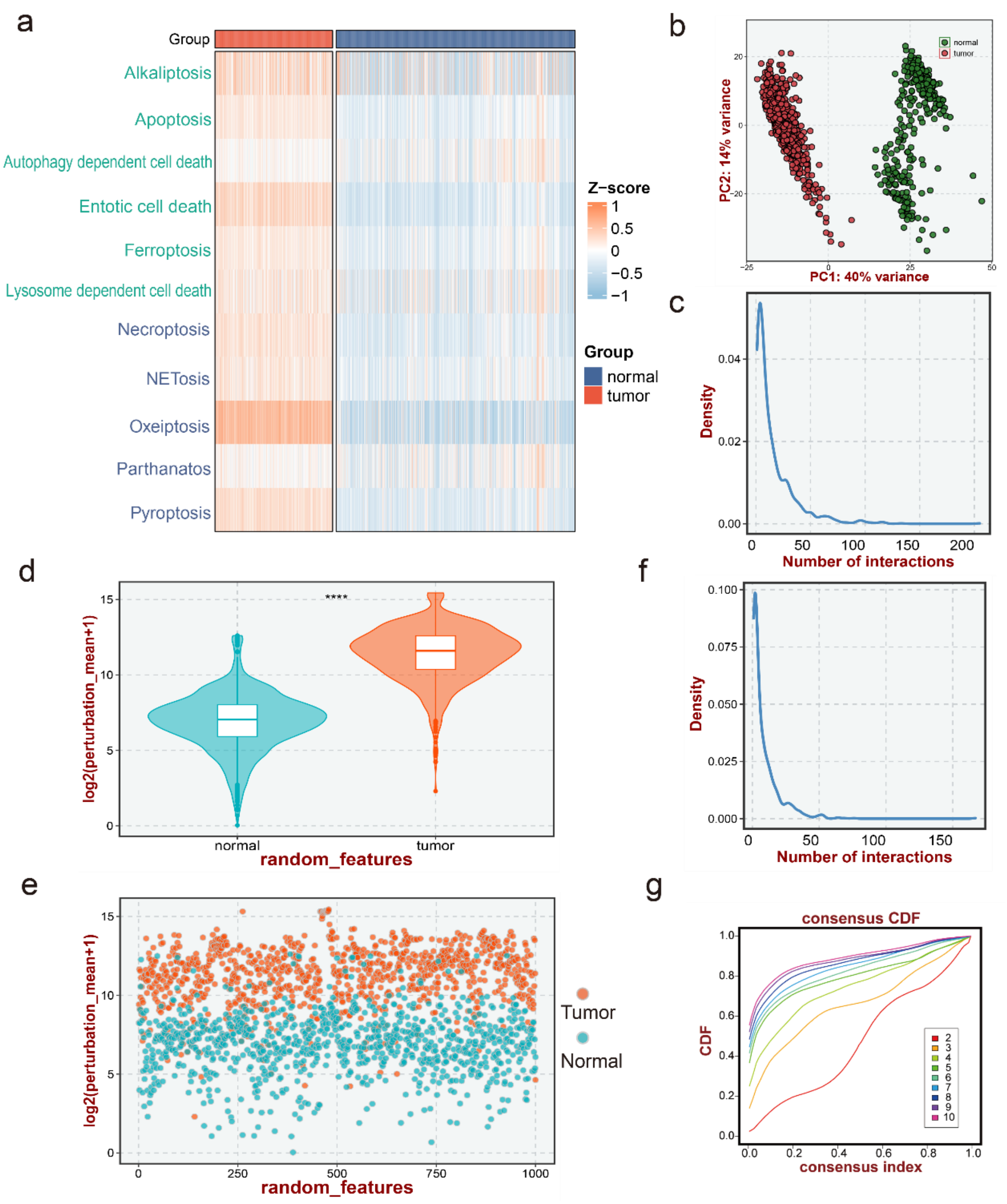

Supplementary Figure 2. Construction of interaction-perturbation in the cell death interplay network. a Differences between colorectal cancer samples and normal tissues in different cell death pathways. **b** PCA (Principal Component Analysis) showed the distribution difference between tumor and normal samples. **c** As the number of interactions increased, the density decreased significantly, presenting a power

distribution in the background networks. R was computed as the Pearson correlation between log10 (interaction number) and log10 (corresponding frequency), which was used to measure the fitting level of the power law curve. The better the curve fitting level is, the closer R is to 1. **d** The distribution of gene interaction perturbations between normal and tumor samples. **e** The scatterplot for the log2-transformed mean of the interaction perturbations in the 1000 randomly selected edges in both normal (blue points) and CRC (red points) tissues. **f** This new network with 1,390 genes and 2,225 interactions also met the scale-free distribution.

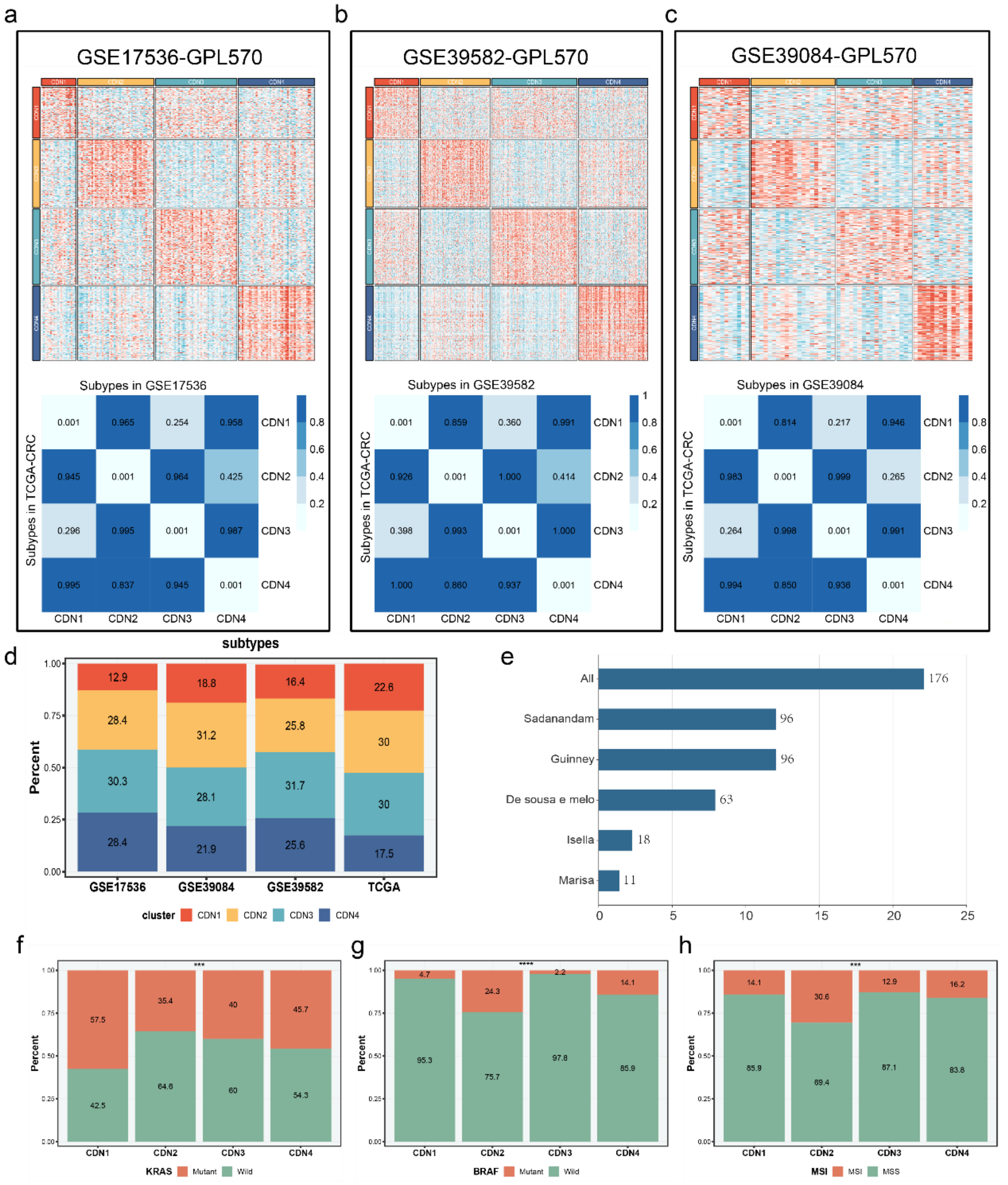

Supplementary Figure 3. Subtype validation of three datasets from the same platform (GPL570). a-c. Three datasets, including GSE17536 (**a**), GSE39582 (**b**), GSE39084 (**c**), were assigned in four subtypes according to the signature genes. Heatmaps and SubMap plots assessed expressive similarity between corresponding subtypes from two different cohorts. **d** Four subtypes also demonstrated analogical proportion in TCGA-CRC and three validation datasets. **e** The proportion of overlap of

our signature genes and the signature genes of previous CRC classifications. **f-h** The proportion of KRAS mutation (**f**), BRAF mutation (**g**), and microsatellite instability (**h**) among different CDN subtypes.

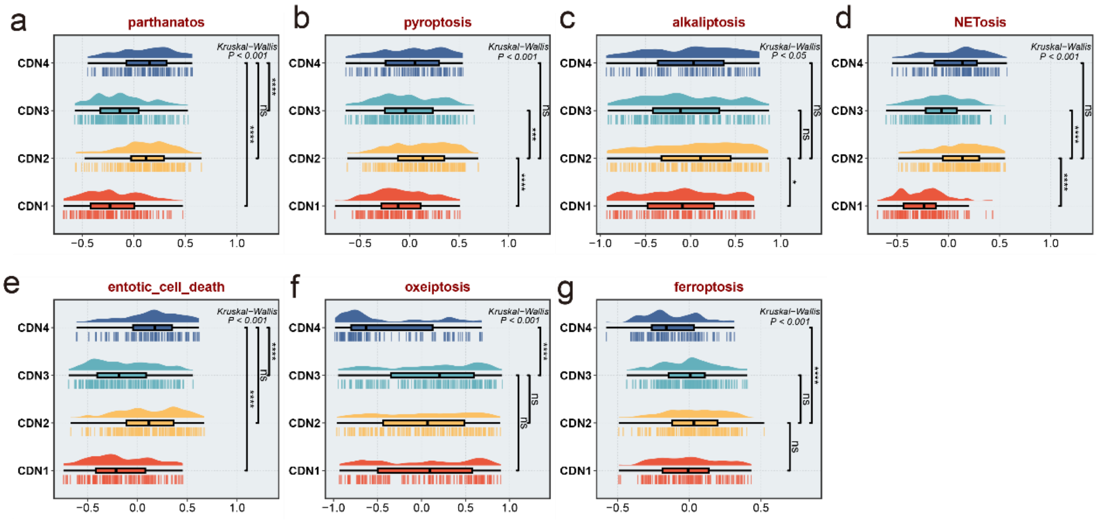

Supplementary Figure 4. Differences in the degree of cell death in CDN subtypes.

a-g The degree of enrichment of four CDN isoforms in seven different cell death pathways. ns fdr > 0.05, *fdr < 0.05, **fdr < 0.01, ***fdr < 0.001, ****fdr < 0.0001.

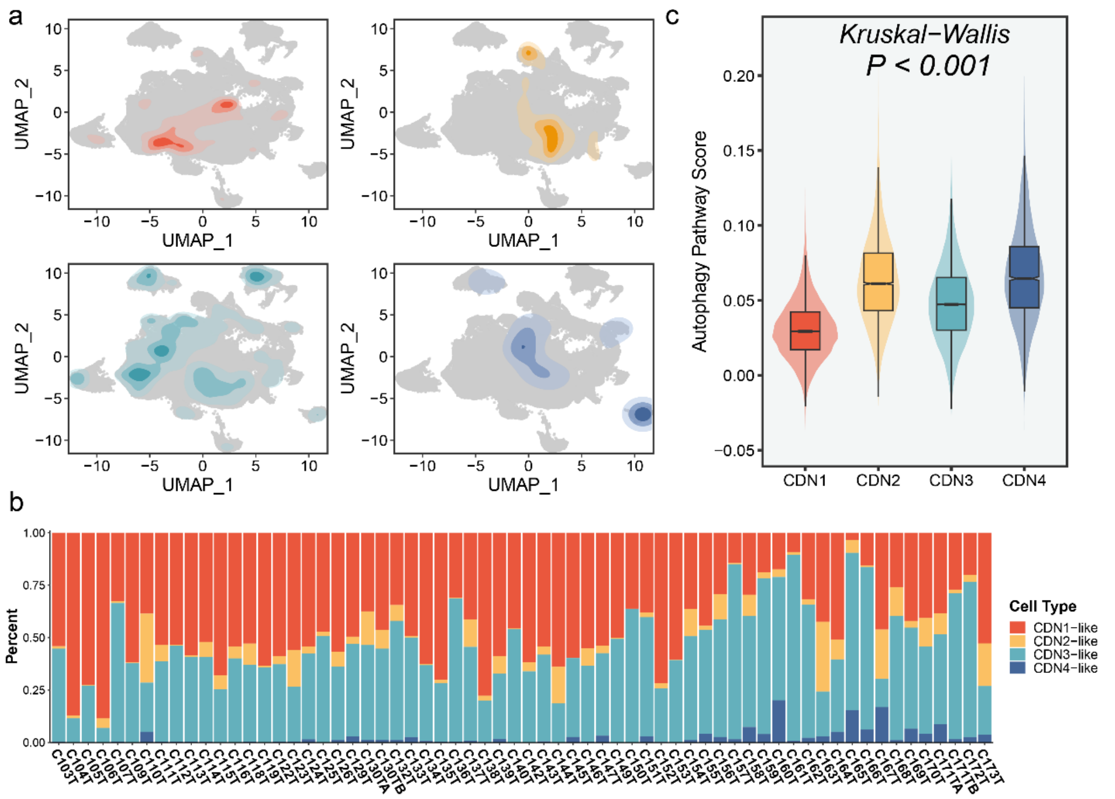

Supplementary Figure 5. Analysis of the distribution differences of CDNs subtypes at the single-cell level. a Spatial distribution of four CDNs-like epithelial cells under UMAP dimensionality reduction analysis. **b** Composition of four CDNs-like epithelial cells at the individual level. **c** Autophagy pathway scores in four CDNs-like epithelial cells were compared by AddModuleScore function calculation.

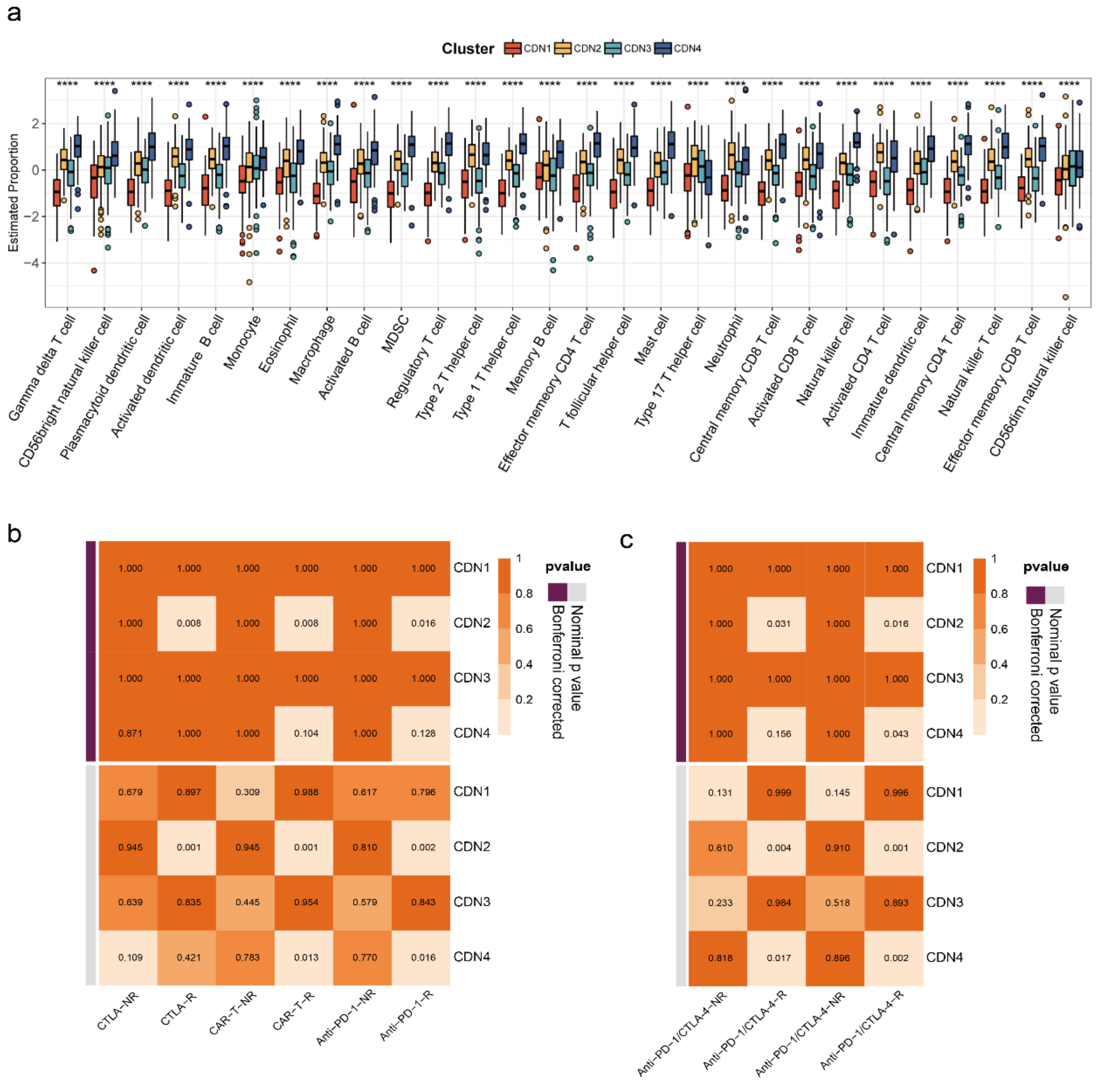

Supplementary Figure 6. Assessment of immune cell infiltration and immunotherapy prediction for CDNs subtypes. a Differential analysis of immune cell infiltration in four CDN subtypes. **b-c** SubMap algorithm evaluated the expression similarity between the four CDN subtypes and the patients with different immunotherapy responses. For SubMap analysis, a smaller p-value implied a more similarity of paired expression profiles. ns fdr > 0.05, *fdr < 0.05, **fdr < 0.01, ***fdr < 0.001, ****fdr < 0.0001.

Supplementary Tables

Supplementary Table 1: Details of baseline information in 4 public datasets

Accession		**TCGA-CRC**	**GSE17536**	**GSE39582**	**GSE39084**
Platform		Illumina RNAseq	Affymetrix Human Genome U133 Plus 2.0 Array		
GPL			GPL570		
PMID			19914252	23700391	25083765
Number of Patients (%)		567 (100%)	165(100%)	521100%)	68 (100%)
Age	≤65	250 (44.1)			
	>65	317 (55.9)			
	Not available				
Gender	Male	311 (54.9)			
	Female	256 (45.1)			
	Not available				
T stage	T1+T2	116 (20.5)			
	T3+T4	449 (79.2)			
	Not available	2 (0.3)			
N stage	N0	319 (56.3)			
	N1+N2	245 (43.2)			
	Not available	3 (0.5)			
M stage	M0	422 (74.4)			
	M1	80 (14.1)			
	Not available	65 (11.5)			
AJCC stage	I+II	301 (53.1)			
	III+IV	246 (43.4)			
	Not available	20 (3.5)			
KRAS	WT	269 (47.4)			
	Mut	208 (36.7)			
	Not available	90 (15.9)			
BRAF	WT	421 (74.2)			
	Mut	56 (9.9)			
	Not available	90 (15.9)			
Microsatellite state	MSI-L/MSS/pMMR	459 (81.0)			
	MSI-H	108 (19.0)			
	Not available				
Survival status	Alive	117 (20.6)			
	Dead	450 (79.4)			
	Not available				
Relapse status	No	202 (35.6)			
	Yes	29 (5.1)			
	Not available	353 (59.3)			

Supplementary Table 2. The details of Indicators for the assessment of immunogenicity and antigen presentation capacity.

Indicator	Details	Reference(s) [PMID]
Nonsilent Mutation Rate	/	https://pubmed.ncbi.nlm.nih.gov/29628290
Silent Mutation Rate	/	https://pubmed.ncbi.nlm.nih.gov/29628290
Wound Healing	The values of Wound Healing reflect the characteristics of "Immune Subtype" C1	https://pubmed.ncbi.nlm.nih.gov/29628290
Proliferation	/	https://pubmed.ncbi.nlm.nih.gov/29628290
Immune Subtype	Using data compiled by TCGA, an extensive immunohistochemical analysis was performed on more than 10000 tumors including 33 different cancer types. In all cancer types, six immune subtypes were identified: Wound Healing, IFN-γ Dominant, Inflammatory, Lymphocyte Depleted, Immunologically Quiet, and TGF-β Dominant.	https://pubmed.ncbi.nlm.nih.gov/29628290
SNV Neoantigens	Single nucleotide variation (SNV) neoantigens were identified through NetMHCpan v3.0,based on HLA types obtained from RNA-seq using OptiType (version 1.2)	https://pubmed.ncbi.nlm.nih.gov/29628290; https://pubmed.ncbi.nlm.nih.gov/27029192; https://pubmed.ncbi.nlm.nih.gov/25143287
Indel Neoantigens	Insertion-deletion (indel) neoantigens were identified through NetMHCpan v3.0,based on HLA types obtained from RNA-seq using OptiType (version 1.2)	https://pubmed.ncbi.nlm.nih.gov/29628290; https://pubmed.ncbi.nlm.nih.gov/27029192; https://pubmed.ncbi.nlm.nih.gov/25143287
CTA score	Cancer/testis-antigen (CTA)	https://pubmed.ncbi.nlm.nih.gov/29628290
Intratumor Heterogeneity	Intratumor genetic heterogeneity (ITH) is a feature of tumors that refers to the repertoire of co-existing genetically distinct subclonal populations.	https://pubmed.ncbi.nlm.nih.gov/29628290; https://pubmed.ncbi.nlm.nih.gov/26840267
Number of Segs	Number of copy number variant segments	https://pubmed.ncbi.nlm.nih.gov/29628290

Fraction Altered	Fraction of genome alterations	https://pubmed.ncbi.nlm.nih.gov/29628290
Homologous Recombination Defects	Homologous recombination defects (HRD) score was determined by three separate DNA-based measures of genomic instability: large (> 15 Mb) non-arm-level regions with loss of heterozygosity (LOH), telomeric allelic imbalance (TAI), and large-scale state transitions (LST) with breaks between adjacent segments of > 10 Mb	https://pubmed.ncbi.nlm.nih.gov/29628290
Aneuploidy score	Aneuploidy scores (AS) were the sum of amplified or deleted (collectively "altered") chromosome arms	https://pubmed.ncbi.nlm.nih.gov/29628290; https://pubmed.ncbi.nlm.nih.gov/29622463
TCR Richness	TCR diversity (Richness) scores were identified using MiTCR v1.0.3, with previously described parameters	https://pubmed.ncbi.nlm.nih.gov/29628290; https://pubmed.ncbi.nlm.nih.gov/23892897; https://pubmed.ncbi.nlm.nih.gov/25196070
TCR Shannon	TCR diversity (Shannon Entropy) scores were identified using MiTCR v1.0.3, with previously described parameters	https://pubmed.ncbi.nlm.nih.gov/29628290; https://pubmed.ncbi.nlm.nih.gov/23892897; https://pubmed.ncbi.nlm.nih.gov/25196070
Number of Segs with LOH	Number of Segs with loss of heterozygosity (LOH)	https://pubmed.ncbi.nlm.nih.gov/29622463
Fraction of Segs with LOH	Fraction of Segs with loss of heterozygosity (LOH)	https://pubmed.ncbi.nlm.nih.gov/29622463

Supplementary Table 3: 30 genes with AUC >0.9 in TCGA and three validation GEO cohorts.

Cohorts	TCGA	GSE17536	GSE39582	GSE39084
AKAP12	0.927134594	0.921171171	0.939456036	0.942857143
AMOTL1	0.935638436	0.960687961	0.958946535	0.97
AOC3	0.935508936	0.906838657	0.919627887	0.958571429
BNC2	0.931990849	0.930896806	0.939106744	0.922857143
CACNA2D1	0.934796685	0.927723178	0.90875326	0.966428571
CALD1	0.92009842	0.949221949	0.961030645	0.944285714
CRYAB	0.908400242	0.929668305	0.933343424	0.964285714
DDR2	0.939696106	0.941441441	0.9585856	0.968571429
DPYSL3	0.930480014	0.951883702	0.955115965	0.941428571
FBXL7	0.918997669	0.909295659	0.930910022	0.954285714
FRMD6	0.929314513	0.947276822	0.943321535	0.944285714
HEG1	0.914680998	0.925982801	0.909801136	0.902857143
IL1R1	0.927091427	0.944512695	0.905877422	0.917142857
JAM3	0.923940257	0.90990991	0.957200075	0.982857143
LAYN	0.914314081	0.917997543	0.920384687	0.945
MGP	0.939178106	0.90509828	0.943542753	0.934285714
MITF	0.914939998	0.914107289	0.946837742	0.92
MPDZ	0.924177674	0.94021294	0.936487053	0.965714286
MSRB3	0.934472934	0.956695332	0.966956967	0.981428571
NAP1L3	0.927091427	0.908476658	0.953823584	0.911428571
PALLD	0.910148493	0.942260442	0.944031762	0.925714286
PEG3	0.90684624	0.927313677	0.943403037	0.942857143
PTPRM	0.908443408	0.915233415	0.923679676	0.955714286
SPOCK1	0.911400328	0.910012285	0.929023845	0.927142857
SSPN	0.908400242	0.928235053	0.931864754	0.925714286
TAGLN	0.915242165	0.951678952	0.940981278	0.914285714
TIMP2	0.913623414	0.944410319	0.940550484	0.97
TNS1	0.930523181	0.927927928	0.944695417	0.97
TSHZ3	0.929400846	0.911445536	0.947117176	0.978571429
TSPYL5	0.93039368	0.920966421	0.929373137	0.908571429
