# Supplementary material for: Crosstalk of cell death pathways unveils an autophagy-related gene AOC3 as a critical prognostic marker in colorectal cancer
Source: Commun Biol. 2024 Mar 9;7:296. doi: 10.1038/s42003-024-05980-6 (PMC10924944; doi:10.1038/s42003-024-05980-6)
Supplement: Supplementary file 2 — Description of Additional Supplementary Files [file 42003_2024_5980_MOESM2_ESM.pdf]

Description of Additional Supplementary Files

File name: Supplementary Data 1
Description: Cell death-related genes.

File name: Supplementary Data 2
Description: 8403 interaction.

File name: Supplementary Data 3
Description: Subtype-specific genes.
