# Supplementary material for: Crosstalk of cell death pathways unveils an autophagy-related gene AOC3 as a critical prognostic marker in colorectal cancer
Source: Commun Biol. 2024 Mar 9;7:296. doi: 10.1038/s42003-024-05980-6 (PMC10924944; doi:10.1038/s42003-024-05980-6)
Supplement: Supplementary file 6 — Reporting Summary [file 42003_2024_5980_MOESM6_ESM.pdf]

nature portfolio

Corresponding author(s): Xinwei Han
Last updated by author(s): Dec 13, 2023

Reporting Summary

Nature Portfolio wishes to improve the reproducibility of the work that we publish. This form provides structure for consistency and transparency in reporting. For further information on Nature Portfolio policies, see our Editorial Policies and the Editorial Policy Checklist.

Statistics

For all statistical analyses, confirm that the following items are present in the figure legend, table legend, main text, or Methods section.

n/a	Confirmed	
☐	☒	The exact sample size (*n*) for each experimental group/condition, given as a discrete number and unit of measurement
☐	☒	A statement on whether measurements were taken from distinct samples or whether the same sample was measured repeatedly
☐	☒	The statistical test(s) used AND whether they are one- or two-sided<br>*Only common tests should be described solely by name; describe more complex techniques in the Methods section.*
☐	☒	A description of all covariates tested
☐	☒	A description of any assumptions or corrections, such as tests of normality and adjustment for multiple comparisons
☐	☒	A full description of the statistical parameters including central tendency (e.g. means) or other basic estimates (e.g. regression coefficient) AND variation (e.g. standard deviation) or associated estimates of uncertainty (e.g. confidence intervals)
☐	☒	For null hypothesis testing, the test statistic (e.g. *F*, *t*, *r*) with confidence intervals, effect sizes, degrees of freedom and *P* value noted<br>*Give P values as exact values whenever suitable.*
☐	☒	For Bayesian analysis, information on the choice of priors and Markov chain Monte Carlo settings
☐	☒	For hierarchical and complex designs, identification of the appropriate level for tests and full reporting of outcomes
☐	☒	Estimates of effect sizes (e.g. Cohen's *d*, Pearson's *r*), indicating how they were calculated

Our web collection on statistics for biologists contains articles on many of the points above.

Software and code

Policy information about availability of computer code

Data collection	Public data used in this work can be acquired from the TCGA (https://portal.gdc.cancer.gov/) and GEO (https://www.ncbi.nlm.nih.gov/geo/).
Data analysis	All data processing, visualization, and statistical analysis were conducted in the R 4.2.1 software.

For manuscripts utilizing custom algorithms or software that are central to the research but not yet described in published literature, software must be made available to editors and reviewers. We strongly encourage code deposition in a community repository (e.g. GitHub). See the Nature Portfolio guidelines for submitting code & software for further information.

Data

Policy information about availability of data

All manuscripts must include a data availability statement. This statement should provide the following information, where applicable:

- Accession codes, unique identifiers, or web links for publicly available datasets
- A description of any restrictions on data availability
- For clinical datasets or third party data, please ensure that the statement adheres to our policy

Public data used in this work can be acquired from the TCGA (https://portal.gdc.cancer.gov/) and GEO (https://www.ncbi.nlm.nih.gov/geo/). Other data supporting the findings of this study are available from the corresponding author upon reasonable request.

Research involving human participants, their data, or biological material

Policy information about studies with human participants or human data. See also policy information about sex, gender (identity/presentation), and sexual orientation and race, ethnicity and racism.

Reporting on sex and gender	Based on public data from previously published studies, we collected information on patients' gender and included in the multivariate Cox regression analysis section demonstrating that classification was an independent prognostic factor. Detailed data sources have already been described in the article.
Reporting on race, ethnicity, or other socially relevant groupings	Because this study was a subtype development based on molecular biology, no socially constructed or socially relevant categorical variables were included in the estimation manuscript.
Population characteristics	Age, Gender, Genotypic information, Past diagnosis and treatment categories.
Recruitment	The criteria for our screening cohort were as follows: (1) primary tumor tissue samples; (2) RNA expression data available; (3) no preoperative chemotherapy or radiotherapy; (4) survival information available and survival time >30 days.
Ethics oversight	Sample data were obtained from public databases and previously published studies.

Note that full information on the approval of the study protocol must also be provided in the manuscript.

Field-specific reporting

Please select the one below that is the best fit for your research. If you are not sure, read the appropriate sections before making your selection.

☒ Life sciences ☐ Behavioural & social sciences ☐ Ecological, evolutionary & environmental sciences

For a reference copy of the document with all sections, see nature.com/documents/nr-reporting-summary-flat.pdf

Life sciences study design

All studies must disclose on these points even when the disclosure is negative.

Sample size	The sample sizes were determined based on previous publications. A total of 1321 patients and 308 normal samples were enrolled from public databases. Table S1 summarizes the data sources and details of this study.
Data exclusions	The criteria for our screening cohort were as follows: (1) primary tumor tissue samples; (2) RNA expression data available; (3) no preoperative chemotherapy or radiotherapy; (4) survival information available and survival time >30 days.
Replication	All attempts at replication were successful. How many times each experiment was performed and which statistical analysis used is indicated in the figure legends.
Randomization	Not available.
Blinding	Not available.

Reporting for specific materials, systems and methods

We require information from authors about some types of materials, experimental systems and methods used in many studies. Here, indicate whether each material, system or method listed is relevant to your study. If you are not sure if a list item applies to your research, read the appropriate section before selecting a response.

Materials & experimental systems

n/a	Involved in the study	
☐	☒	Antibodies
☒	☐	Eukaryotic cell lines
☒	☐	Palaeontology and archaeology
☒	☐	Animals and other organisms
☒	☐	Clinical data
☒	☐	Dual use research of concern
☒	☐	Plants

Methods

n/a	Involved in the study	
☒	☐	ChIP-seq
☒	☐	Flow cytometry
☒	☐	MRI-based neuroimaging

Antibodies

Antibodies used	AOC3 antibody (1:600; Cat no. 66834-1-Ig, Proteintech, Wuhan, China) was used in this study.
Validation	The validation of the antibody can be accessed by this website: https://ptgcn.com/products/AOC3-Antibody-66834-1-Ig.htm#product-information.

Plants

Seed stocks	Not available.
Novel plant genotypes	Not available.
Authentication	Not available.
